# The amount of C1q–adiponectin complex is higher in the serum and the complex localizes to perivascular areas of fat tissues and the intimal–medial layer of blood vessels of coronary artery disease patients

**DOI:** 10.1186/s12933-015-0209-0

**Published:** 2015-05-09

**Authors:** Eun Shil Hong, Cheong Lim, Hye Yeon Choi, Eu Jeong Ku, Kyoung Min Kim, Jae Hoon Moon, Soo Lim, Kyong Soo Park, Hak Chul Jang, Sung Hee Choi

**Affiliations:** Department of Internal Medicine, Seoul National University College of Medicine and Seoul National University Bundang Hospital, 300, Gumi-dong, Bundang-gu, Seongnam, 463-707 South Korea; Department of Internal Medicine, Seoul National University College of Medicine, Seoul, South Korea; Department of Thoracic and Cardiovascular Surgery, Seoul National University College of Medicine and Seoul National University Bundang Hospital, Seongnam, South Korea

**Keywords:** C1q, Adiponectin, High-molecular weight adiponectin, Coronary artery disease, Biomarker

## Abstract

**Background:**

The complement component C1q triggers activation of the classical immune pathway and can bind to adiponectin (APN). Recently, some studies have been reported that serum C1q-APN/total APN ratio correlates with atherosclerosis and coronary artery disease (CAD). We assessed the relationships between C1q related variables and the severity of CAD, and investigated the localization of the C1q–APN complex.

**Methods:**

The sample included 153 subjects comprising healthy controls and patients with subclinical or overt CAD. We measured the serum concentrations of C1q, total APN, and high-molecular weight (HMW)-APN, and the amount of C1q–APN complex. We identified the sites of C1q–APN complex deposition in various adipose tissues and blood vessels.

**Results:**

Serum concentrations of C1q and HMW-APN and the C1q/HMW-APN ratio were independently associated with the severity of coronary stenosis. The amount of C1q–APN complex was significantly higher in patients with CAD compared with controls. C1q and APN co-localized in perivascular areas of subcutaneous, visceral, and pericardial fat tissues, and the internal mammary artery of patients with severe CAD.

**Conclusions:**

Serum C1q concentration and the C1q/HMW-APN ratio were independent markers of coronary artery stenosis. The amount of C1q–APN complex was significantly greater in serum from CAD patients. C1q and APN co-localized to perivascular areas in adipose tissue and blood vessels. The association between the increased amount of the C1q–APN complex and CAD should be investigated further.

## Background

The incidence of cardiovascular diseases has increased markedly in recent years, and atherosclerotic coronary artery disease (CAD) is now the most common cause of death in the world. The process of atherosclerosis is mediated by chronic inflammation in which dyslipidemia, hypertension, and diabetes play important pathogenic roles [1,2]. Considering the potentially fatal consequences of CAD, it is important to find biomarkers that can predict CAD early and precisely. To date, a number of adipokines and inflammatory cytokines have been investigated in the search for reliable predictors of CAD [3,4].

Adiponectin (APN) is a well-known adipokine secreted almost exclusively by adipocytes and circulates at a high concentration [5]. It has beneficial effects on insulin sensitivity and glucose homeostasis [6,7]. APN also inhibits inflammation and plays a role in the prevention of atherosclerosis. Thus, it is considered to be a protective adipokine and is therefore a potential therapeutic agent to prevent or treat atherosclerosis [8,9]. APN has a adhesive property and exists as large homo-oligomers in trimer, hexamer, and high-molecular weight (HMW) forms [10]. Some studies have suggested that the HMW form (HMW-APN) is the most biologically active form [11] and is a more accurate, independent risk factor for CAD than is the total APN amount or concentration [12,13].

Complement is a major system involved in host innate immunity [14]. Complement 1q (C1q), a subcomponent of complement C1, plays a key role in triggering activation of the classical pathway by recognition of immune complexes [15]. Like APN, C1q is abundant in the circulation and is structurally similar to APN; both have a collagen-like domain and a globular heads domain [16]. C1q is important in the immune response involving complement activation, but little is known about its role in chronic, low-grade systemic inflammation, which plays an important role in metabolic diseases such as atherosclerosis. One study has demonstrated that recombinant and native human APN binds to purified C1q under physiological conditions [17]. Recent studies have reported that the C1q–APN protein complex exists in human blood [18,19] and that the serum C1q–APN/total APN ratio may serve as a biomarker of CAD [20]. However, the clinical significance and physiological function of the C1q–APN complex and the tissue sites of C1q–APN complex deposition are not well known.

We measured the amount of serum APN parameters including C1q, total APN, HMW-APN, and C1q–APN complex to determine whether any of these variables correlates with the severity of coronary artery stenosis and could thus serve as a biomarker of CAD. We identified the deposition sites of C1q and APN in various adipose tissues and blood vessel to provide a better understanding of the C1q–APN complex in CAD patients.

## Methods

### Study subjects

A total of 153 subjects were enrolled in this study. This included 113 asymptomatic patients who had undergone a cardiac evaluation with 64-slice multidetector computed tomography (MDCT) either as a health check or for cardiac evaluation of high-risk patients with prediabetes or drug-naïve patients with type 2 diabetes at the Seoul National University Bundang Hospital [SNUBH] from March 2005 to May 2010.

The other 40 subjects were symptomatic patients with coronary artery stenosis ≥50% in three major coronary arteries. Elective coronary artery bypass surgery was performed and samples of whole adipose tissue (subcutaneous, visceral (preperitoneal), and/or pericardial fat), internal mammary artery, and blood were obtained as a part of a study to identify candidate biomarkers of atherosclerosis.

We identified their risk factors from medical history, demographics, baseline clinical profile, and concomitant medications. This study was conducted according to the Declaration of Helsinki and was approved by ethics committees of SNUBH (SNUBH IRB#B-1203/147-006, #A111218-CP02) and all subjects provided their written informed consent.

### Measurement of anthropometric and biochemical parameters

Body weight, height, and blood pressure were measured at enrollment in the study. Body mass index was calculated as the weight in kilograms divided by the square of the height in meters (kg/m^2^). Serum concentrations of total cholesterol, triglycerides, high-density lipoprotein-cholesterol, low-density lipoprotein (LDL)-cholesterol, aspartate aminotransferase, alanine aminotransferase, creatinine, glycated hemoglobin (HbA1c), high-sensitivity C-reactive protein (hs-CRP), and plasma glucose were measured after a ≥12-hour fast. We defined diabetes mellitus as a fasting plasma glucose concentration of ≥126 mg/dL, an HbA1c level ≥6.5%, or taking antidiabetic medicine. Hypertension was defined as systolic blood pressure >140 mmHg, or diastolic blood pressure >90 mmHg, or taking antihypertensive medicine. Dyslipidemia was defined as an LDL-C concentration of ≥130 mg/dL, or hypertriglyceridemia (triglyceride concentration ≥200 mg/dL), or taking lipid-lowering medicine. Serum C1q concentration was measured using an enzyme-linked immunosorbent assay (ELISA) kit (HK356, Hycult Biotech, Uden, The Netherlands) using samples that had been frozen at −80°C until analyzed. ELISA kits were used to measure serum total APN concentration (EZHADP-61 K, Millipore, Billerica, MA, USA) and HMW-APN (EZHMWA-64 K, Millipore) in the same samples.

### MDCT protocol

CAD was detected using 64-slice MDCT during a routine health examination. Subjects with a heart rate >70 beats per min received 10–30 mg of intravenous esmolol (Jeil Pharm, Seoul, Korea) before MDCT imaging. CT angiography was performed with a 64-slice MDCT scanner (Brilliance 64, Philips Medical Systems, Best, The Netherlands) using the standard scanning protocol described previously [21].

### Definition of coronary artery stenosis

Coronary artery stenosis was identified as percent of luminal narrowing of at least 1 major coronary artery and was evaluated with MDCT or coronary angiography. Coronary artery stenosis was categorized into three classifications: normal (any coronary artery stenosis <25%), mild to moderate (any coronary artery stenosis ≥25%), and severe (three coronary artery stenosis ≥50%; coronary artery bypass surgery). The presence of CAD was defined as any coronary artery stenosis ≥50% regardless of number of involved coronary artery.

### Blood and tissue samples

Serum was obtained from peripheral blood at the time of patient enrollment. Fat tissues from subcutaneous, visceral, and pericardial areas, and samples of the internal mammary artery were obtained from 40 patients during coronary artery bypass surgery and were irrigated with saline. All of the samples were stored at −80°C until analyzed.

### Immunoblotting assay to detect the C1q–APN complex

Human serum was mixed with C1q antibody (SC-53544, Santa Cruz Biotechnology, Dallas, TX, USA) in 1× kinase buffer (20 mM HEPES pH 7.4, 5 mM MgCl_2_, 1 mM dithiothreitol) and agitated overnight at 4°C. Protein G (17-0618-01, GE Healthcare, Buckinghamshire, UK) was added to the mixture, and the mixture was rotated for 4 h at 4°C. After three washes, the C1q-immunocomplexes were subjected to sodium dodecyl sulfate-polyacrylamide gel electrophoresis and western blotting using anti-APN antibody (ab18851, Abcam, Cambridge, UK).

### Histological analysis

Deparaffinized adipose tissue sections and blood vessels were fixed in 4% paraformaldehyde in phosphate-buffered saline. The sections of fat tissues and vessels were stained with hematoxylin–eosin. Immunostaining was performed overnight at 4°C with mouse anti-C1q (ab71089, Abcam,) or with rabbit anti-APN (NB100-65810, Novus Biologicals, Littleton, CO, USA) antibodies. The sections were then washed and incubated in Alexa Fluor 488 goat anti-rabbit IgG (Moleaulr Probes, Eugene, OR) and Alexa Fluor 594 goat anti-mouse IgG (Molecular Probes, Eugene, OR) as the secondary antibody for 1 h at 37°C. The slides were examined under a Zeiss Axio Imager A1 fluorescent microscope (Carl Zeiss, Cambridge, UK).

### Statistical analysis

All values are expressed as mean and standard deviation (SD) or number (percent). Clinical parameters that were not normally distributed were log-transformed to reduce the skewness; we present the results as the anti-logarithm for ease of interpretation. Differences in parameters between groups were analyzed using *χ*^2^-test, Student’s *t*-test, or one-way analysis of variance (ANOVA). When significantly different values were observed in an ANOVA, Bonferroni post hoc analysis was applied to identify the significantly different values. Simple and multivariate logistic regression analysis was used to determine the associations between parameters and CAD. A *p*-value <0.05 was considered significant. All analyses were performed using SPSS 17.0 for Windows.

## Results

### Baseline characteristics of subjects

The characteristics of the study subjects are summarized in Table 1. Serum C1q and HMW-APN concentrations were significantly lower in patients with severe coronary artery stenosis compared with those with no stenosis or mild to moderate stenosis. There was a trend toward a stepwise decrease in these concentrations with severity of stenosis. However, serum total APN concentration did not differ significantly between the groups. The C1q/HMW-APN ratio was significantly higher in patients with severe stenosis compared with those with no stenosis or with mild to moderate stenosis (Table 1).

**Table 1:** **Baseline characteristics of subjects according to coronary artery stenosis**

	**Normal (n = 72)**	**Mild to moderate (n = 41)**	**Severe (n = 40)**	***P*** **value**
Age (years)	56.4 ± 7.8^a^	57.4 ± 11.0^a^	67.0 ± 10.3^b^	<0.001
Sex (male/female)	36/36^a^	32/9^b^	31/9^b^	0.002
Body mass index (kg/m^2^)	24.9 ± 2.7^a,b^	25.9 ± 2.5^a^	24.1 ± 3.4^b^	0.025
Systolic blood pressure (mmHg)	128.1 ± 13.7	126.5 ± 13.4	125.1 ± 17.7	0.596
Diastolic blood pressure (mmHg)	77.2 ± 10.1^a^	78.7 ± 9.3^a^	70.7 ± 10.3^b^	0.001
Fasting blood glucose (mg/dL)	114.4 ± 32.3^a^	129.9 ± 42.4^a,b^	156.4 ± 70.0^b^	0.001
HbA1c (%)	6.3 ± 1.1^a^	6.9 ± 1.6^a,b^	7.0 ± 1.5^b^	0.018
Total cholesterol (mg/dL)	215.3 ± 31.2^a^	209.3 ± 39.7^a^	171.0 ± 49.4^b^	<0.001
Triglycerides (mg/dL)	172.7 ± 99.6	206.2 ± 201.3	147.5 ± 110.9	0.205
HDL-cholesterol (mg/dL)	52.8 ± 12.0^a^	48.7 ± 12.5^a,b^	43.4 ± 10.2^b^	<0.001
LDL-cholesterol (mg/dL)	118.7 ± 27.5	120.2 ± 36.5	101.4 ± 37.9	0.039
Aspartate aminotransferase (IU/L)	24.6 ± 11.3^a^	26.2 ± 8.3^a^	37.7 ± 22.4^b^	<0.001
Alanine aminotransferase (IU/L)	30.3 ± 21.2	35.5 ± 22.8	29.2 ± 18.3	0.225
Creatinine (mg/dL)	1.0 ± 0.2	1.1 ± 0.2	1.2 ± 0.7	0.265
hs-CRP (mg/dL)	0.1 ± 0.1^a^	0.6 ± 2.2^a,b^	2.0 ± 4.5^b^	<0.001
Diabetes mellitus (%)	38.9	48.8	62.5	0.056
Sulfonyl ureas/biguanides/thiazolidinediones/dipeptidyl peptidase-4 inhibitors/insulin (n)	2/6/1/1/0	3/7/2/2/1	8/8/4/2/2	0.912
Hypertension (%)	30.6^a^	61.0^b^	75.0^b^	<0.001
Calcium channel blockers/angiotensin receptor blockers/angiotensin-converting-enzyme inhibitor/β-blockers/diuretics (n)	6/5/1/1/1	10/12/2/3/5	12/13/6/4/4	0.934
Dyslipidemia (%)	51.4	63.4	72.5	0.081
Statins/fibrates/ezetimibe (n)	18/3/0	20/4/0	25/5/1	0.821
C1q (μg/ml)	5.77 ± 1.68^a^	5.38 ± 1.34^a^	4.13 ± 0.75^b^	<0.001
Total APN (μg/ml)	7.74 ± 4.59	5.89 ± 4.23	6.51 ± 3.95	0.097
HMW-APN (μg/ml)	4.91 ± 3.70^a^	3.19 ± 2.20^a^	1.51 ± 0.50^b^	<0.001
C1q/Total APN ratio	1.15 ± 0.98	1.67 ± 1.52	1.00 ± 0.85	0.48
C1q/HMW-APN ratio	2.46 ± 2.52^a^	2.77 ± 2.33^a,b^	3.08 ± 1.31^b^	0.047

Data are presented as the mean ± SD. ^a,b^Values with the same letter did not differ significantly by Bonferroni post hoc analysis or *χ* ^2^-tests. Normal, any coronary artery stenosis <25%; mild to moderate, any coronary artery stenosis ≥25%; severe, three coronary artery stenosis ≥50% (coronary artery bypass surgery). HbA1c, glycated hemoglobin; HDL, high-density lipoprotein; LDL, low-density lipoprotein; hs-CRP, high-sensitivity C-reactive protein; APN, adiponectin; HMW-APN, high-molecular weight APN.

Men had significantly lower serum concentrations of both total APN and HMW-APN than women: 5.40 ± 3.71 and 9.51 ± 4.17 μg/mL (*P* <0.001) for total APN and 2.91 ± 2.33 and 5.34 ± 3.75 μg/mL for HMW-APN (*P* <0.001), respectively. Serum C1q concentration did not differ significantly between men and women (5.19 ± 1.50 and 5.32 ± 1.64 μg/mL, respectively; *P* = 0.656).

### Simple and multivariate logistic regression analyses of the associations between APN parameters and CAD

Logistic regression analysis was performed to evaluate the relationship between CAD and various APN parameters (Table 2). Simple logistic regression analysis showed that the presence of CAD correlated significantly with the concentrations of C1q, HMW-APN, and the C1q/HMW-APN ratio. Following adjustment for age and sex, the associations between CAD and the concentration of C1q, HMW-APN, and the C1q/HMW-APN ratio were significant. After adjusting for age, sex, body mass index, diabetes mellitus, hypertension, and dyslipidemia, the concentrations of C1q total APN, HMW-APN, and the C1q/HMW-APN ratio were independently associated with CAD (odds ratio, OR 0.01 [confidence interval, CI 0.001 – 0.091]; OR 0.88 [CI 0.77 0– 0.996]; OR 0.25 [CI 0.121 – 0.517]; and OR 2.09 [CI 1.122 – 3.907], respectively).

**Table 2:** **Simple and multivariate logistic regression analysis of the associations between APN parameters and CAD**

	**Unadjusted**			**Age and sex adjusted**			**Multivariable adjusted**		
	**OR**	**CI**	***p*** **-value**	**OR**	**CI**	***p*** **-value**	**OR**	**CI**	***p*** **-value**
C1q	**0.02**	**0.003 - 0.090**	<0.001	**0.02**	**0.004 – 0.152**	<0.001	**0.01**	**0.001 – 0.091**	<0.001
Total APN	0.95	0.874 – 1.036	0.252	0.93	0.832 – 1.037	0.191	**0.88**	**0.770 – 0.996**	0.044
HMW-APN	**0.35**	**0.210 – 0.581**	<0.001	**0.31**	**0.165 – 0.573**	<0.001	**0.25**	**0.121 – 0.517**	<0.001
C1q/total APN	0.84	0.537 – 1.327	0.464	0.89	0.517 – 1.546	0.689	0.98	0.521 – 1.826	0.937
C1q/HMW-APN	**1.77**	**1.143 – 2.733**	0.010	**1.99**	**1.151 – 3.430**	0.014	**2.09**	**1.122– 3.907**	0.020

OR, odds ratio; CI, confidence interval; APN, adiponectin; HMW-APN, high-molecular weight APN.

Multivariable analysis adjusted for age, sex, body mass index, diabetes, hypertension, and dyslipidemia.

Bold means significant value.

### Detection of C1q-APN complex in human serum

**Figure 1:**
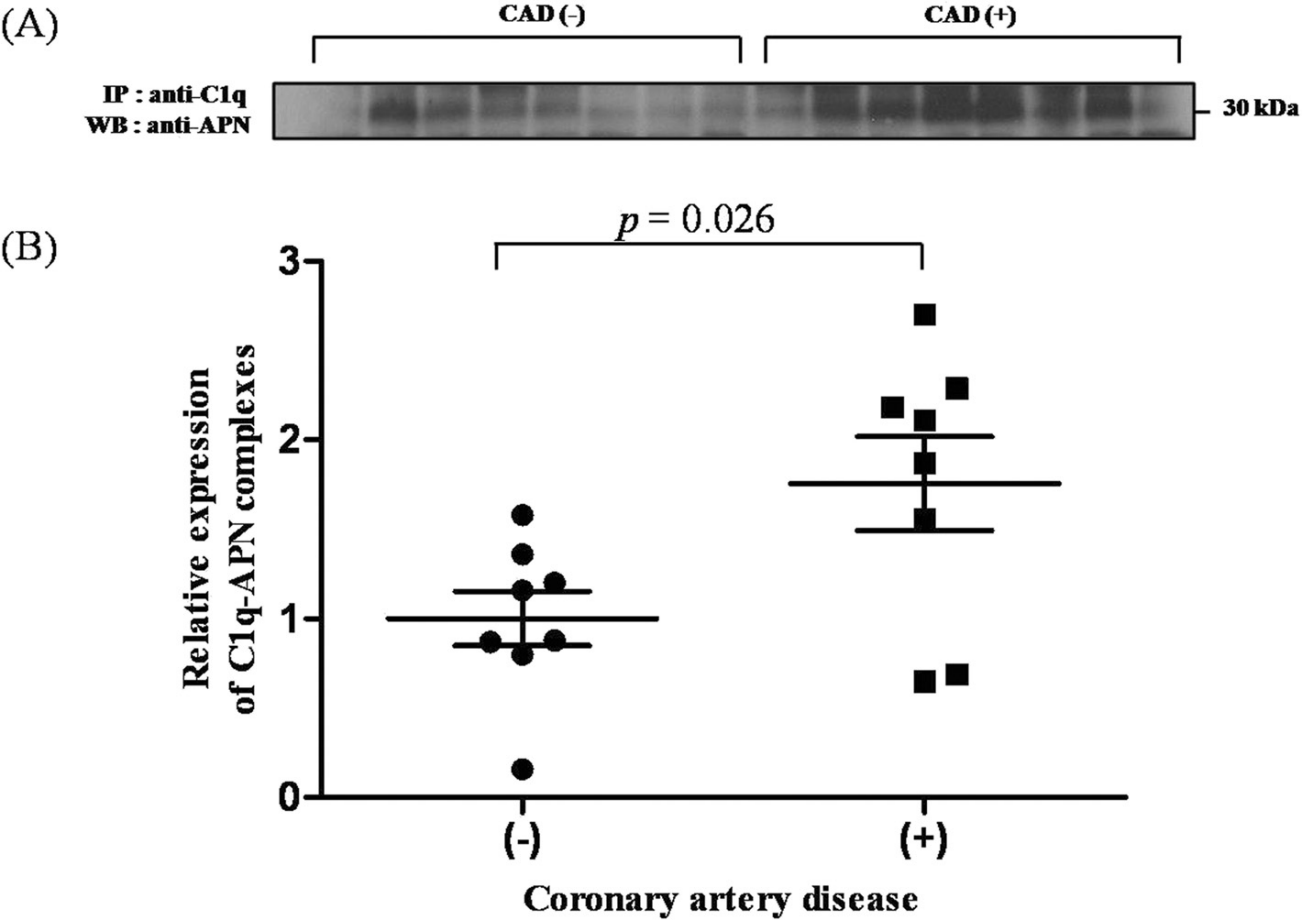
Detection of the C1q–APN complex in human serum. **A**, The amount of C1q–APN complex was measured by coimmunoprecipitation of the serum from 16 subjects. **B**, The relative expression of the C1q–APN complex was 1.8-times higher in patients with CAD (CAD(+)) than in normal controls (CAD(−)) (*P* = 0.026).

To confirm whether C1q and APN form complexes in human serum, we performed immunoprecipitation and an immunoblotting assay. Serum samples from normal controls without CAD (n = 8) and from patients with CAD (n = 8) were subjected to immunoprecipitation with antibody against C1q, followed by immunoblotting with antibody against APN. APN was detected in C1q immunoprecipitates (Figure 1A). The serum C1q–APN complex was detected, and the relative amount of serum C1q–APN complex in the serum was about twofold higher in patients with CAD compared with the controls (*P* = 0.026) (Figure 1B).

### Immunofluorescence of C1q and APN in adipose tissue and blood vessels

**Figure 2:**
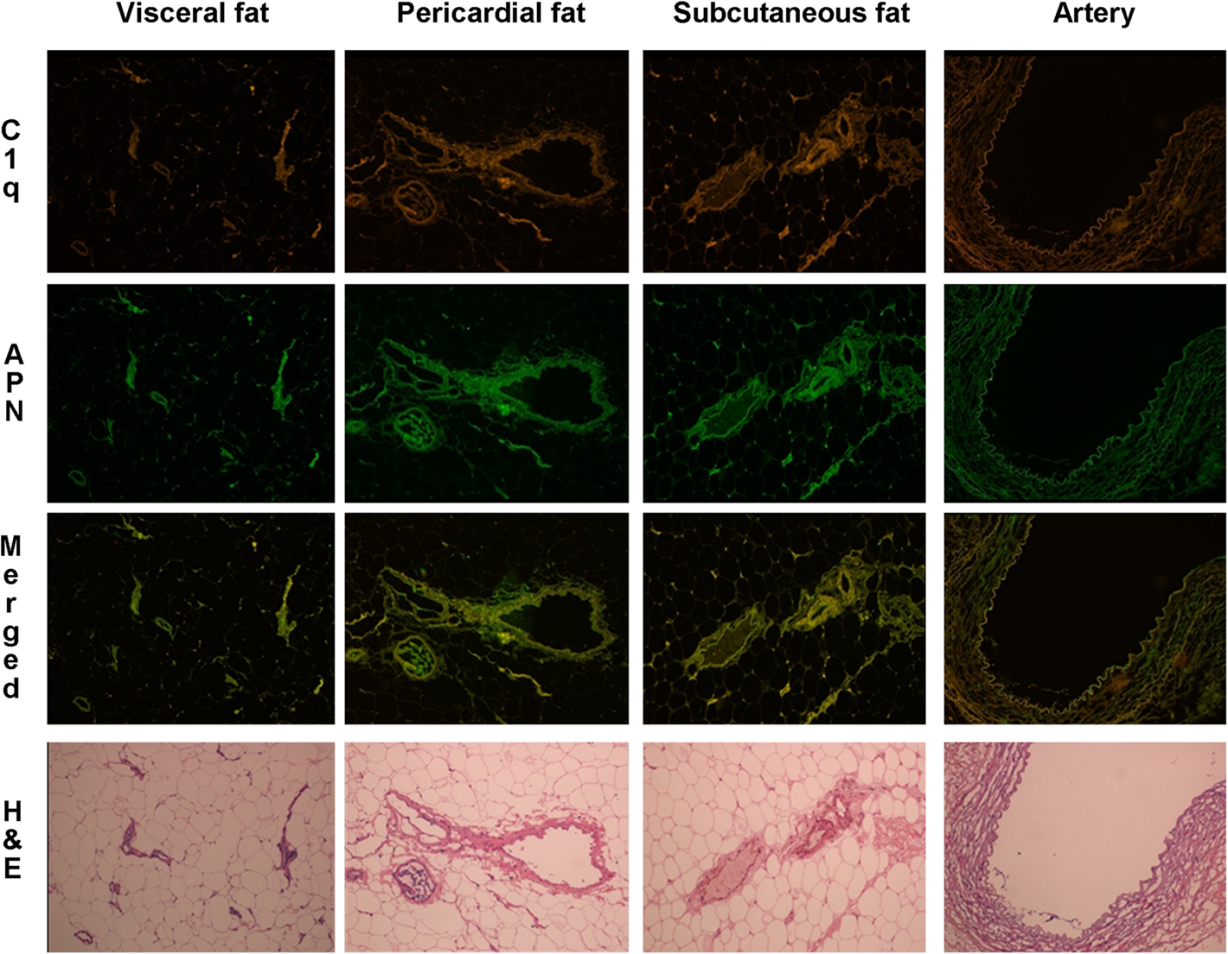
Co-localization of C1q and APN deposited in adipose tissue and blood vessels (×100). Positive staining of C1q (red) and APN (green) is shown along the perivascular areas of fat tissues and intimal–medial layer of the blood vessel. C1q and APN co-localized almost completely in the same areas. H&E, hematoxylin and eosin.

We selected samples of adipose tissue (visceral, pericardial, and subcutaneous fat) and the internal mammary artery taken during coronary artery bypass surgery from four patients with CAD. The samples were stained for C1q and total APN using immunofluorescent antibodies. Positive staining for C1q and total APN by immunofluorescence was shown along the perivascular areas in all fat tissues and in the intimal–medial layer of blood vessels. C1q and APN were almost completely co-localized in the same areas. Because the results were similar among 4 subjects, we showed the results of 1 subject, representatively (Figure 2).

## Discussion

The major findings of the present study are as follows. First, serum C1q concentration was significantly lower and the serum C1q/HMW-APN ratio significantly higher in subjects with severe coronary artery stenosis compared with subjects with no stenosis or mild to moderate stenosis. By contrast, APN concentration did not differ according to the presence or absence of stenosis. Second, the amount of the C1q–APN complex in serum was significantly higher in subjects with CAD than in subjects without CAD. Third, the C1q and APN co-localized to the perivascular areas of adipose tissues and intimal–medial layer of blood vessels of CAD patients.

C1q is the first component of the serum complement system. It comprises six globular “heads” linked via six collagen-like “stalks” to a fibril-like central region and possesses significant homology to APN [16]. The traditionally accepted role of C1q is the recognition of immune complexes and activation of the classical pathway. Binding of Fc regions of immunoglobulin to the globular head portion of C1q induces further activation of the cascade of proteins comprising the classical pathway [22]. C1q is produced by and deposits around macrophages and dendritic cells, and promotes phagocytosis [23]. However, the role of C1q is not restricted to recognition of immune complexes or other molecules. Nontraditional roles of C1q are rapidly emerging and include autoimmune diseases and carcinogenesis [24], but little is known about its possible role in chronic metabolic diseases such as atherosclerosis, obesity, and diabetes.

C1q has recently been suggested to play a role in atherosclerosis when complexed with APN. Previously, it has reported that the C1q–APN complex exists in the blood and has suggested that the C1q–APN/total APN ratio is a useful predictive marker of the metabolic syndrome and CAD in Japanese people [18,20,25]. A high serum C1q–APN/total APN ratio was associated with CAD prevalence, independent of other CAD risk factors in a previous study [20]. We measured the serum concentrations of C1q, total APN, and HMW-APN, and their associations with the severity of CAD. Previous studies have reported nonsignificant correlations between C1q, HMW-APN, and atherosclerotic disease [20,25]. However, in contrast to the previous results, we found that the serum concentrations of C1q and HMW-APN were independently associated with CAD and decreased significantly with increasing severity of coronary artery stenosis. The total APN concentration and the C1q/total APN ratio were not associated with the presence or severity of CAD. We could not measure the absolute concentration of the C1q–APN complex in the blood because no commercial ELISA kit is available for the direct measurement of the complex. Instead, we measured the quantity of C1q–APN complex in coimmunoprecipitation experiments. The amount of C1q–APN complex was significantly higher in the blood of patients with CAD than in the controls with no CAD. Our result is concordant with the other study that serum C1q-APN levels were significantly higher in the acute coronary syndrome than in the normal coronary group [26]. Recently, the protein family C1q/TNF-related protein (CTRP) has been discovered as adiponectin paralog. Some CTRP family members have metabolic regulatory function in glucose and/or fat homeostasis [27]. CTRP-3 is an anti-inflammatory adipokine and serum CTRP-3 concentrations are significantly decreased in patients with acute coronary syndrome [28]. Serum CTRP-1 and CTRP-3 levels were positively associated with atherosclerosis [29,30]. These results suggest that APN parameters including C1q, C1q-APN complex and CTRPs might be important in the regulation of atherosclerosis and development of acute coronary syndrome.

Interestingly, we confirmed the deposition of the C1q and APN in human white adipose tissues and blood vessels. Immunofluorescence showed that C1q and APN co-localized in the perivascular areas of fat tissues and in the intimal–medial area of the artery. APN is known to bind to collagen types I, II, and V, which are present in the vascular intima. APN is detected in the walls of injured arteries and in atherosclerotic lesions [31]. Our study is the first to show the co-localization of C1q and APN in human tissues. However, it remains unclear which forms of circulating APN forms protein complex with C1q. The precise physiological role of the complex is also unknown, but we speculate that APN may play a protective role in the C1q-induced activation of the complement pathway, which is known as a cause of vascular damage in autoimmune disease or other types of inflammation that contribute to systemic atherosclerosis. APN is thought to prevent inflammation and to play a beneficial role in preventing atherosclerosis and metabolic syndrome [32,33]. One study reported that adiponectin protects against activation of C1q-induced inflammation in injured tissues of mice [34], which is similar evidence as our result of C1q-APN co-localization in perivascular tissues. Further research is needed to clarify the role of the C1q–APN complex in protecting against the development of atherosclerosis in humans.

The present study has several limitations. First, it was a cross-sectional study, making it difficult to interpret whether there is a causal relationship between the markers measured in this study and CAD. Further prospective studies are needed to confirm this relationship. Second, we could not directly measure the serum concentration and tissue deposit of C1q-APN complex because of the lack of a commercially available method. Third, the number of serum and tissue samples used in the tests for detection of C1q and APN was small. Although we repeated the tests, there could be a selection bias. Forth, we could not get tissue and blood vessel samples of normal healthy control.

## Conclusion

In conclusion, the present study demonstrated that serum C1q concentration and the C1q/HMW-APN ratio were independent markers of coronary artery stenosis. The co-localization of the C1q and APN was observed in the perivascular areas of fat tissues and in the intimal–medial area of the internal mammary artery in conjunction with a higher serum amount of the C1q–APN complex in patients with CAD. These results suggest that the C1q–APN complex may play a role in the development of atherosclerosis. Further human studies are needed to clarify whether the C1q–APN complex is a potential target for new treatments to protect against atherosclerosis.

## Abbreviations

CAD: Coronary artery disease
APN: Adiponectin
HMW: High-molecular weight
C1q: Complement 1q
MDCT: Multidetector computed tomography
LDL: Low-density lipoprotein
HbA1c: Glycated hemoglobin
hs-CRP: High-sensitivity C-reactive protein
ELISA: Enzyme-linked immunosorbent assay
SD: Standard deviation
ANOVA: Analysis of variance
OR: Odds ratio
CI: Confidence interval
CTRP: C1q/TNF-related protein
